# Myeleterosis in an ALPS5 patient with primary immune dysregulation syndrome

**DOI:** 10.1111/cns.13301

**Published:** 2020-03-17

**Authors:** Hongyu Long, Zhao Chen, Bo Xiao, Beisha Tang, Hong Jiang

**Affiliations:** ^1^ Department of Neurology Xiangya Hospital Central South University Changsha China; ^2^ National Clinical Research Centre for Geriatric Diseases Xiangya Hospital Central South University Changsha China; ^3^ Key Laboratory of Hunan Province in Neurodegenerative Disorders Central South University Changsha China; ^4^ Laboratory of Medical Genetics Central South University Changsha China

**Keywords:** autoimmune lymphoproliferative syndrome, CTLA4, immune dysregulation, myeleterosis

Dear Editor,

Autoimmune lymphoproliferative syndrome type V (ALPS5) is an autosomal dominant immune dysregulation syndrome characterized by autoimmunity and multiorgan lesions including lungs, and gastrointestinal tract and brain. Mutation in the *CTLA4* gene leads to a broad clinical syndrome with incomplete penetrance.^1^, ^2^ Immunosuppressive therapies including sirolimus and abatacept are often implemented.^2^, ^3^, ^4^ The *CTLA4* deficiency‐associated overactive T cell and exhausted B cell contribute to multisystem lymphocytic infiltration and hypogammaglobulinemia.^1^, ^2^, ^5^ Here, we described an ALPS5 patient with spinal cord involvement rather than brain, which has been rarely reported.

A 36‐year‐old man was admitted to our department complaining of progressive weakness and numbness of both lower limbs accompanying with urinary incontinence for 2 years and blurred vision for 2 weeks. He had a history of Evans syndrome and refractory pulmonary fungal infection. Neurological examination at admission found impaired ambulation. Diffusely brisk tendon reflexes in the lower limbs and bilateral Hoffman's and Babinski's signs were observed. Deep and superficial sensibilities under T10 level were declined.

Laboratory investigations showed normal blood count and vitamin B12 level, and a HIV‐negative result, whereas low immunoglobulin levels (IgG < 0.33 g/L, IgA < 66.7 mg/L, and IgM < 41.7 mg/L). The anti‐AQP4, MOG, and MBP antibody detection in serum and CSF showed no abnormality. The anterior segment, vitreous, and retina of ophthalmic examination showed no inflammation. VEP record showed prolonged p100 latency. Lymphopenia was remarkable with low CD4+ T cells and low CD 19+ B cells. Flow cytometry showed reduced levels of CD19+ B cells and impaired T‐cell function. The proportion of CD45RA+ CD62L+ naïve CD4+ T cells and CD25+ Treg cells (CD4+CD25+FOXP3+) was lower (Figure 1A). MRI scan showed increased T2‐weighted signals and mild speckled enhancement in spinal cord without abnormality in brain (Figure 2).

**Figure 1:**
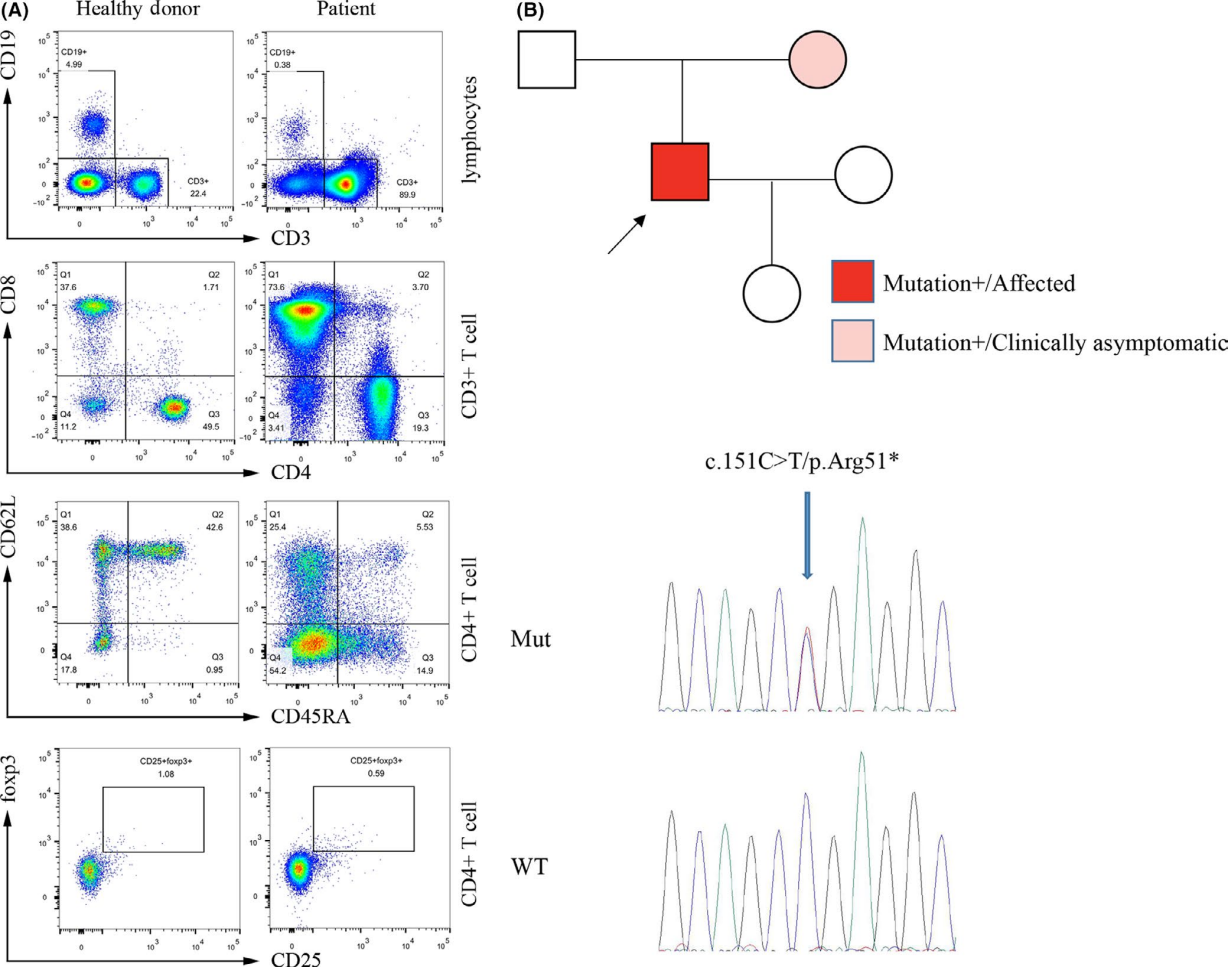
Mutation and flow cytometry result of the family. Flow cytometry showed reduced levels of CD19^+^ B cells and inverted CD4/CD8 ratio. The proportion of CD45RA^+^CD62L^+^ naïve CD4^+^ T cells and CD25^+^ Treg cells was lower in the patient than in the healthy donor (A). Symbols filled in red and pink represented proband and his unaffected mother. Chromatograms of the *CLTA4* gene showed the heterozygous c.151C > T(p.Arg51*) variation in proband (B)

**Figure 2:**
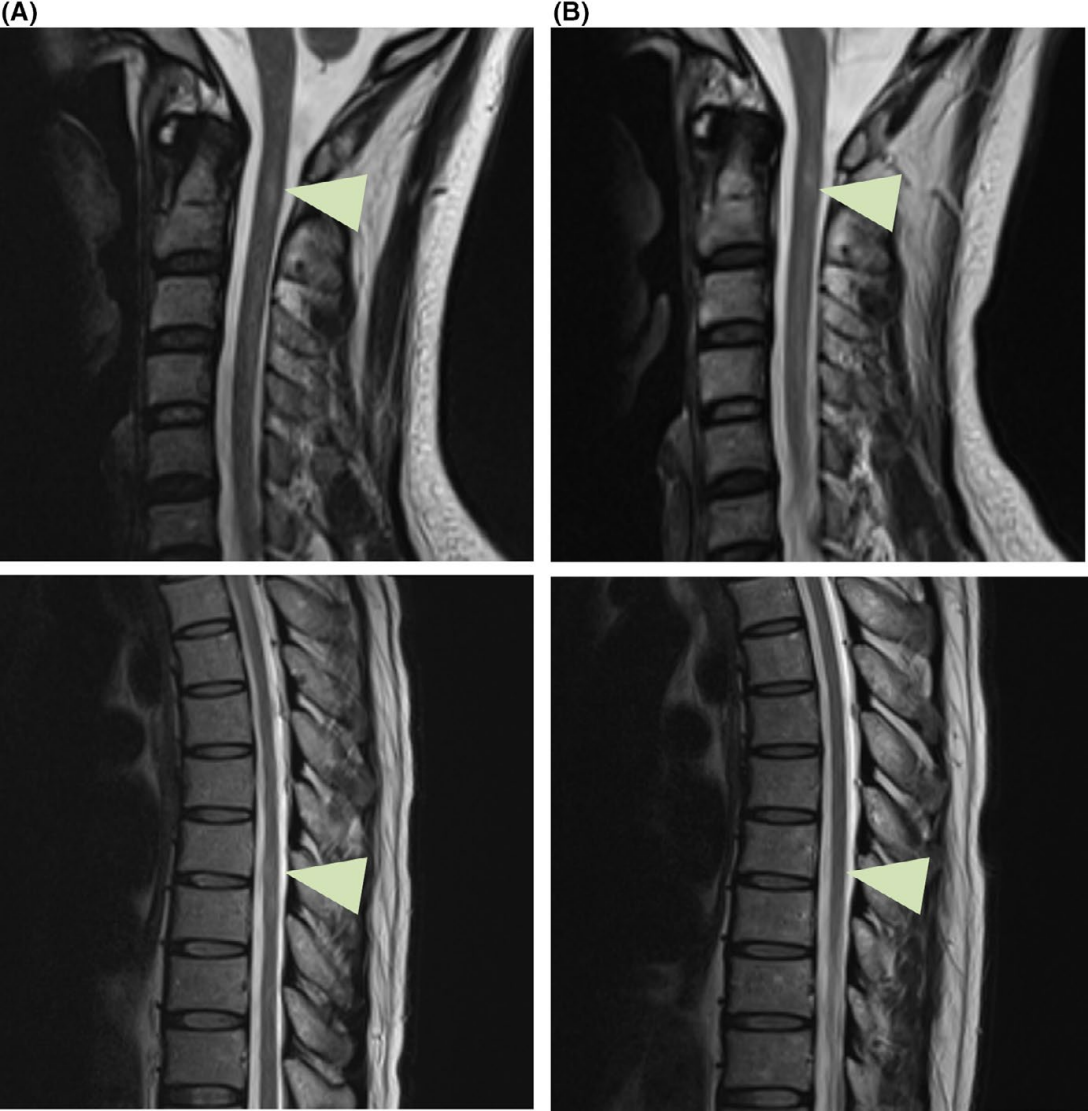
Myeleterosis. MRI scan of spinal cord showed hyperintensity in C2 and T8/9 level (A). A follow‐up MRI scan showed the abnormality of spinal cord had narrowed (B)

Whole‐exome sequencing identified a heterozygous nonsense variation of c.151C > T(p.Arg51*) in the *CTLA4* gene in the proband and his mother (Figure 1B). Thus, the patient was diagnosed with ALPS5 and treated with sirolimus and IVIG for 6 months. Although harboring the same heterozygous mutation, his mother was apparently healthy with normal flow cytometry result, suggesting the incomplete penetrance. A follow‐up visit showed that the hyperintensity in T2‐weighted MRI of patient had narrowed. His vision was recovered, but weakness of lower limbs and urinary incontinence were still present without marked development.

This study illustrates the first report of myeleterosis associated with *CTLA4* deficiency in the progression of ALPS5. Clinically, with the exclusion of the subacute combined degeneration and tumor of spinal cord, visual impairment and spinal cord involvement with T2‐weighted hyperintensity and mild speckled enhancement in our case may apparently be attributed to NMO spectrum disorders (NMOSD). However, the genetic finding of c.151C > T variation and immune tests finally confirmed the causality that is due to autoimmune lymphoproliferation rather than demyelination. Because sirolimus inhibited the damage of T lymphocyte infiltration on spinal cord, controlled trend of progressive symptoms and alleviated MRI features of myeleterosis were observed. Thus, ALPS5 may be the immunosuppressant‐responsive to some extent. Nevertheless, previous serious damage of spinal cord was difficult to fully recover as a complication of long nature history, which requires further treatment.

The *CTLA4* haploinsufficiency results in highly variable features of autoimmunity.

Till now, the lymphocytic infiltration of nonlymphoid organs has been reported in intestine, lung, bone marrow, kidney, and central nervous system. In the latter, only brain lesion is reported. Interestingly, previous report showed that the patient carrying the variation of c.151C > T developed brain lymphocytic infiltrates with complete penetrance,^2^ whereas our case firstly observed myeleterosis in addition to other multiorgan lesions, which unveils the multiple neurological involvements across brain and spinal cord, and broadens the phenotypic spectrum associated with *CTLA4* mutation.

This case highlights atypical features of ALPS5 clinical phenotype. The improved myeleterosis has been observed to be partially immunosuppressant‐responsive after treated with sirolimus, pointing to beneficial effect of immunosuppressant in therapeutic interventions. Additionally, as a major complication in progression of ALPS5, severe infection often contributes to a bad prognosis and requires immunoregulation and antiinfection treatments.

Overall, the varied phenotype and penetrance shed new light on clinically and genetically heterogeneous manifestations in such primary immune dysregulation syndrome, suggesting that other genetic or environmental factors implicated in key pathogenesis may contribute to phenotypic variability. Thus, our case demonstrates a new neurological feature in ALPS5 that myeleterosis should not be underestimated and deserves full attention.

## CONFLICT OF INTEREST

The authors declare no conflict of interest.
